# Supplementary material for: Cost-Effectiveness of Screening Algorithms for Familial Hypercholesterolaemia in Primary Care
Source: J Pers Med. 2022 Feb 22;12(3):330. doi: 10.3390/jpm12030330 (PMC8953997; doi:10.3390/jpm12030330)
Supplement: Supplementary file 1 [file jpm-12-00330-s001.zip › jpm-1531618-supplementary.pdf]

Cost-effectiveness of screening algorithms for familial hypercholesterolemia in primary care Appendices

List of Supplementary

Supplementary

Table S1: Key design criteria for economic model

Table S2: Model inputs for the decision tree

Table S3: Scenario analysis for different thresholds associated with FAMCAT1 and FAMCAT2 algorithms

Table S4: Results of scenario analysis for different thresholds associated with FAMCAT1 and FAMCAT2 algorithms

Table S5: Results of scenario analysis for a different proportion of patients who have VUS within the population (3 in 100)

Table S6: Sensitivity analysis of proportion of patients with no relevant mutation being identified as above risk by the algorithms

Table S7: Consolidated Health Economic Evaluation Reporting Standards (CHEERS) Statement

Table S1: Key design criteria for economic model.

**Decision problems**	Which algorithm is cost-effective for the screening of electronic health records to identify index patients with a FH mutation within primary-care
**Model type**	Decision tree built in Microsoft Excel
**Population**	A hypothetical cohort of 4,500 adult patients (mean age of 56 years) registered at NHS primary care practices in England eligible for electronic screening of health records to detect possible FH.
**Setting and perspective**	Primary care and the healthcare service in NHS England
**Time Horizon**	12-weeks[a]
**Costs**	National currency (£) at 2018/2019 prices
**Consequences**	Clinical effectiveness measured as the number of patients who were correctly identified via genetic testing as having a monogenic mutation for FH.
**Discounting**	Not required due to assumed time horizon set at less than one-year
**Analytical strategy**	1) All interventions were ranked by expected total cost, from cheapest to most expensive.<br>2) Incremental total cost and incremental cases identified were estimated with each intervention being compared to the next cheapest alternative.<br>3) Remove any dominated interventions (comparator has the same/increased effectiveness and/or is the same/cheaper total cost) and re-estimate incremental total cost and cases identified.<br>4) Estimate the incremental cost per additional FH case identified ratio (ICER) using the following formulae:<br>$$ICER = \frac{Total\ Cost_{Intervention} - Total\ Cost_{Comparator}}{Cases\ Identified_{Intervention} - Cases\ Identified_{Comparator}}$$<br>5) Remove any extendedly dominated interventions (occurs if the ICER for the intervention decreases compared with the previous ICER), then re-estimate incremental total cost, total cases identified, and ICERs.
[a] Data from the FAMCAT feasibility study suggested that it took a mean time of 12 weeks from the electronic health record being screened to when a patient received their genetic test result.	
[b] Microsoft Corporation. *Microsoft Excel for Microsoft 365*. Available from: https://www.microsoft.com/en-gb/microsoft-365/excel.	

Table S2: Model inputs for the decision tree.

**Model input**	**Mean**	**95% CI**		**Distribution fitted**	**Source**	**Notes**
Proportion of patients with a particular characteristic						
FH monogenic mutation (Node A)	0.0037	0.0026	0.0051	Beta	Wald et al [7]	
Variant of Unknown Significance (VUS) (Node C)	0.0021	0.0017	0.0025	Beta	*	95% CI from assumed standard error of 10% of mean value
Proportion of patients which algorithm identifies as above risk given patient has monogenic FH (Node B)						
FAMCAT1	0.3125	0.1416	0.5560	Beta	FAMCAT	
FAMCAT2	0.6875	0.4440	0.8584	Beta	FAMCAT	
Dutch-Lipid	0.3750	0.1848	0.6136	Beta	FAMCAT	
Cholesterol	0.4375	0.2310	0.6682	Beta	FAMCAT	
Simon-Broome	0.7778	0.4526	0.9368	Beta	FAMCAT	
No active screening	0	0	0	N/A		
Proportion of patients which algorithm identifies as above risk given patient has VUS (Node D)						
FAMCAT1	0.0000	0.0000	0.2991	Normal†	FAMCAT	
FAMCAT2	0.0000	0.0000	0.2991	Normal†	FAMCAT	
Dutch-Lipid	0.0000	0.0000	0.2991	Normal†	FAMCAT	
Cholesterol	0.0000	0.0000	0.2991	Normal†	FAMCAT	
Simon-Broome	0.3333	0.1206	0.6458	Beta	FAMCAT	
No active screening	0	0	0	N/A		
Proportion of patients which algorithm identifies as above risk given patient has no relevant mutation (Node E)						
FAMCAT1	0.0553	0.0326	0.0923	Beta	FAMCAT	
FAMCAT2	0.0553	0.0326	0.0923	Beta	FAMCAT	
Dutch-Lipid	0.0468	0.0263	0.0819	Beta	FAMCAT	
Cholesterol	0.0723	0.0457	0.1128	Beta	FAMCAT	
Simon-Broome	0.2894	0.2351	0.3504	Beta	FAMCAT	
No active screening	0	0	0	N/A		
Costs per patient associated with the screening of electronic health records (1st stage)						
Initial electronic health record search	£0.00				FAMCAT	
GP review of electronic health record search	£0.00				FAMCAT	
Mail merge for patients identified as above risk by electronic algorithm for request to submit Family History Questionnaire	£0.00				FAMCAT	
Posting of Family History Questionnaire	£0.83				FAMCAT	

Entry of Family History Questionnaire	£1.16				FAMCAT	
Second electronic health record search	£0.00				FAMCAT	
GP review of second health record search	£0.00				FAMCAT	
Mail merge for patients identified as above risk by electronic algorithm to invite for genetic test	£0.00				FAMCAT	
Total cost per individual patient for 1st stage screening	£2.00	£1.61	£2.39	Gamma		95% CI from assumed standard error of 10% of mean value
Costs per patient associated with genetic testing within primary care (2nd stage)						
Invitation for genetic test	£1.16				FAMCAT	
Postage for invitation	£0.83				FAMCAT	
Phlebotomy for blood test	£7.00				FAMCAT	
Blood test consumables	£1.50				FAMCAT	
Blood test postage and packing	£1.95				FAMCAT	
Genetic test	£278.00				FAMCAT	
Total cost per individual patient for 2nd stage screening	£290.44	£233.51	£347.37	Gamma		95% CI from assumed standard error of 10% of mean value
Cost per patient of letter to inform genetic test results if no relevant mutation detected (3rd stage)						
Letter to patient with no genetic mutation to inform genetic tests results	£1.16				FAMCAT	
Postage for above results letter	£0.83				FAMCAT	
Total cost per individual patient for 3rd stage screening	£1.99	£1.60	£2.38	Gamma		95% CI from assumed standard error of 10% of mean value
Cost per patient to inform patient of genetic test results if test confirms monogenic FH or VUS (3rd stage)						
Invite patient for consultation if genetic test confirms monogenic FH	£1.16				FAMCAT	
Postage for above results letter	£0.83				FAMCAT	
GP consultation	£78.00				Curtis and Burns[a]	Assumed double visit
Total cost per individual patient for 3rd stage screening	£79.99	£64.31	£95.67	Gamma		95% CI from assumed standard error of 10% of mean value
*Expert opinion from the FAMCAT steering committee suggested that the proportion of VUS was estimated as 9/16ths of the proportion of monogenic FH, based upon FAMCAT trial data as 16 patients had monogenic FH, while an additional nine had VUS.						
†The Beta distribution could not be fitted to these inputs due to the mean value being zero. To allow uncertainty, we used a confined normal distribution to prevent sampling values less than zero and greater than one.						
[a] *Unit Costs of Health and Social Care 2019*. Avaliable from https://www.pssru.ac.uk/project-pages/unit-costs/unit-costs-2019/						

Table S3: Scenario analysis for different thresholds associated with FAMCAT1 and FAMCAT2 algorithms.

**Threshold**	**Proportion of patients with monogenic FH who are identified as above risk by algorithm (node B)**	**Proportion of patients with VUS who are identified as above risk by algorithm (node D)**	**Proportion of patients with no relevant mutation who are identified as above risk by algorithm (node E)**
FAMCAT 1			
0.002	1.0000	0.7778	0.8723
0.004	1.0000	0.5556	0.7617
0.010	1.0000	0.4444	0.5915
0.050	0.6875	0.0000	0.1830
0.080	0.5000	0.0000	0.1021
0.100	0.3750	0.0000	0.0681
0.145*	0.3125	0.0000	0.0553
0.150	0.3125	0.0000	0.0468
0.200	0.1875	0.0000	0.0255
0.250	0.1250	0.0000	0.0128
FAMCAT 2			
0.002	0.7500	0.2222	0.2766
0.0036	0.6875	0.0000	0.1064
0.004	0.6875	0.0000	0.0766
0.0047*	0.6875	0.0000	0.0553
0.010	0.5000	0.0000	0.0085
0.050	0.1875	0.0000	0.0000
0.100	0.1250	0.0000	0.0000

* Threshold at which algorithm is using for initial analysis

Table S4: Results of scenario analysis for different thresholds associated with FAMCAT1 and FAMCAT2 algorithms.

Threshold	Expected total cost per patient (£)	Number of monogenic FH cases identified	Number of genetic tests to find one case	Incremental cost (£)	Incremental monogenic FH cases identified	ICER per additional monogenic FH case identified (£)	Notes
**FAMCAT1**							
0.002	257.00	16	238	169.72	3.6630	208,506	Vs Simon-Broome
0.004	224.83	16	208	205.81	5.1511	179,796	Vs FAMCAT2
0.010	175.34	16	162	156.32	5.1511	136,561	Vs FAMCAT2
0.050	56.14	11	73	37.12	0.0000	Dominated	Dominated by FAMCAT2
0.080	32.36	8	56	13.35	-3.0907	Dominated	Dominated by FAMCAT2
0.100	22.31	6	50	3.29	-5.1511	Dominated	Dominated by FAMCAT2
0.145*	18.50	5	49	2.38	-1.0302	Dominated	Dominated by Dutch-Lipid
0.150	16.03	5	42	16.03	5.1511	14,007	VS no active screening, extendedly dominated by FAMCAT2
0.200	9.68	3	38	9.68	3.0907	14,091	VS no active screening, extendedly dominated by FAMCAT2
0.250	5.88	2	29	5.88	2.0604	12,845	VS no active screening, extendedly dominated by FAMCAT2
**FAMCAT2**							
0.002	83.44	12	101	59.81	5.1511	52,253	Vs cholesterol, extendedly dominated by Simon-Broome
0.0036	33.87	11	43	17.75	5.1511	15,507	Vs Dutch-Lipid
0.004	25.20	11	31	25.20	11.3324	10,008	Vs no active screening, extendedly dominates Dutch-Lipid, dominates Cholesterol
0.0047*	19.01	11	23	19.01	11.3324	7,549	Vs no screening, extendedly dominates Dutch-Lipid, dominates Cholesterol
0.010	5.15	8	6	5.15	8.2418	2,814	Vs no active screening, dominates Dutch-Lipid and Cholesterol
0.050	2.25	3	1	2.25	3.0907	3,282	Vs no active screening
0.100	2.17	2	1	2.17	2.0604	4,738	Vs no active screening

*=Initial analysis threshold level

Table S5: Results of scenario analysis for a different proportion of patients who have VUS within the population (3 in 100).

Screening algorithm	Expected total cost per patient (£)	Number of FH cases identified	Number of genetic tests to find one FH case	Incremental cost (£)	Incremental number of FH cases identified	Incremental cost per additional FH case identified	Notes
No active screening	0	0	-	-	-	-	
Dutch-Lipid	16.15	6	35	16.15	6.1813	11,754	Vs no active screening, extendedly dominated by FAMCAT2
FAMCAT1	18.54	5	49	2.39	-1.0302	Dominated	Dominated by Dutch-Lipid
FAMCAT2	19.05	11	23	19.05	11.3324	7,564	Vs no active screening
Cholesterol	23.67	7	46	4.62	-4.1209	Dominated	Dominated by FAMCAT2
Simon-Broome	87.36	13	102	68.31	1.4881	206,570	Vs FAMCAT2

Table S6: Sensitivity analysis of proportion of patients with no relevant mutation being identified as above risk by the algorithms.

	**FAMCAT 1**		**FAMCAT 2**		**Dutch-Lipid**		**Cholesterol**		**Simon-Broome**		**ICERS**	
**Percentage of value at Node E**	**E(Cost) per patient (£)**	**Number of tests to identify 1 patient**	**E(Cost) per patient (£)**	**Number of tests to identify 1 patient**	**E(Cost) per patient (£)**	**Number of tests to identify 1 patient**	**E(Cost) per patient (£)**	**Number of tests to identify 1 patient**	**E(Cost) per patient (£)**	**Number of tests to identify 1 patient**	**FAMCAT 2 vs No Active Screening (£)**	**Simon-Broome vs FAMCAT 2 (£)**
0%	2.42	1	2.93	1	2.51	1	2.59	1	3.15	1	*	*
10%	4.03	6	4.54	3	3.87	4	4.70	5	11.56	11	1,803	21,226
20%	5.64	11	6.15	5	5.23	8	6.80	10	19.97	21	2,442	41,804
30%	7.25	15	7.76	8	6.59	11	8.90	14	28.39	31	3,081	62,383
40%	8.86	20	9.37	10	7.95	15	11.01	19	36.80	41	3,719	82,961
50%	10.47	25	10.98	12	9.31	18	13.11	23	45.21	52	4,358	103,539
60%	12.07	30	12.58	14	10.67	21	15.21	28	53.63	62	4,997	124,117
70%	13.68	35	14.19	16	12.04	25	17.32	32	62.04	72	5,636	144,696
80%	15.29	39	15.80	18	13.40	28	19.42	37	70.45	82	6,274	165,274
90%	16.90	44	17.41	21	14.76	31	21.52	41	78.87	92	6,913	185,852
100%	18.51	49	19.02	23	16.12	35	23.63	46	87.28	102	7,552	206,431
110%	20.12	54	20.63	25	17.48	38	25.73	50	95.69	112	8,190	227,009
120%	21.73	59	22.23	27	18.84	42	27.83	55	104.11	122	8,829	247,587
130%	23.33	63	23.84	29	20.20	45	29.94	59	112.52	132	9,468	268,165
140%	24.94	68	25.45	32	21.56	48	32.04	64	120.94	142	10,106	288,744
150%	26.55	73	27.06	34	22.92	52	34.14	68	129.35	153	10,745	309,322
160%	28.16	78	28.67	36	24.28	55	36.25	73	137.76	163	11,384	329,900
170%	29.77	83	30.28	38	25.65	59	38.35	77	146.18	173	12,023	350,478
180%	31.38	87	31.88	40	27.01	62	40.45	82	154.59	183	12,661	371,057
190%	32.98	92	33.49	42	28.37	65	42.56	86	163.00	193	13,300	391,635
200%	34.59	97	35.10	45	29.73	69	44.66	91	171.42	203	13,939	412,213

*=Simon-Broome extendedly dominates all other algorithms with an ICER of £1,105

Table S7: Consolidated Health Economic Evaluation Reporting Standards (CHEERS) Statement.

**Section/item**	**Item No**	**Recommendation**	**Reported on page No/ line No**
**Title and abstract**			
Title	1	Identify the study as an economic evaluation or use more specific terms such as "cost-effectiveness analysis", and describe the interventions compared.	1
Abstract	2	Provide a structured summary of objectives, perspective, setting, methods (including study design and inputs), results (including base case and uncertainty analyses), and conclusions.	1
**Introduction**			
Background and objectives	3	Provide an explicit statement of the broader context for the study.	1-2
		Present the study question and its relevance for health policy or practice decisions.	1-2
**Methods**			
Target population and subgroups	4	Describe characteristics of the base case population and subgroups analysed, including why they were chosen.	2-3, Table A1
Setting and location	5	State relevant aspects of the system(s) in which the decision(s) need(s) to be made.	3, Table A1
Study perspective	6	Describe the perspective of the study and relate this to the costs being evaluated.	Table A1
Comparators	7	Describe the interventions or strategies being compared and state why they were chosen.	3, Table 1 p15
Time horizon	8	State the time horizon(s) over which costs and consequences are being evaluated and say why appropriate.	Table A1
Discount rate	9	Report the choice of discount rate(s) used for costs and outcomes and say why appropriate.	Table A1
Choice of health outcomes	10	Describe what outcomes were used as the measure(s) of benefit in the evaluation and their relevance for the type of analysis performed.	Table A1
Measurement of effectiveness	11a	*Single study-based estimates:* Describe fully the design features of the single effectiveness study and why the single study was a sufficient source of clinical effectiveness data.	N/A
	11b	*Synthesis-based estimates:* Describe fully the methods used for identification of included studies and synthesis of clinical effectiveness data.	Table A1, Table A2
Measurement and valuation of	12	If applicable, describe the population and methods used to elicit preferences for outcomes.	N/A

preference-based outcomes			
Estimating resources and costs	13a	*Single study-based economic evaluation:* Describe approaches used to estimate resource use associated with the alternative interventions. Describe primary or secondary research methods for valuing each resource item in terms of its unit cost. Describe any adjustments made to approximate to opportunity costs.	N/A
	13b	*Model-based economic evaluation:* Describe approaches and data sources used to estimate resource use associated with model health states. Describe primary or secondary research methods for valuing each resource item in terms of its unit cost. Describe any adjustments made to approximate to opportunity costs.	Table A1, Table A2
Currency, price date, and conversion	14	Report the dates of the estimated resource quantities and unit costs. Describe methods for adjusting estimated unit costs to the year of reported costs if necessary. Describe methods for converting costs into a common currency base and the exchange rate.	Table A1
Choice of model	15	Describe and give reasons for the specific type of decision-analytical model used. Providing a figure to show model structure is strongly recommended.	3
Assumptions	16	Describe all structural or other assumptions underpinning the decision-analytical model.	3-4
Analytical methods	17	Describe all analytical methods supporting the evaluation. This could include methods for dealing with skewed, missing, or censored data; extrapolation methods; methods for pooling data; approaches to validate or make adjustments (such as half cycle corrections) to a model; and methods for handling population heterogeneity and uncertainty.	5, Table A1
**Results**			
Study parameters	18	Report the values, ranges, references, and, if used, probability distributions for all parameters. Report reasons or sources for distributions used to represent uncertainty where appropriate. Providing a table to show the input values is strongly recommended.	Table A2
Incremental costs and outcomes	19	For each intervention, report mean values for the main categories of estimated costs and outcomes of interest, as well as mean differences between the comparator groups. If applicable, report incremental cost-effectiveness ratios.	6, Table 2 p6
Characterising uncertainty	20a	*Single study-based economic evaluation:* Describe the effects of sampling uncertainty for the estimated incremental cost and incremental effectiveness parameters, together with the impact of methodological assumptions (such as discount rate, study perspective).	N/A

	20b	*Model-based economic evaluation:* Describe the effects on the results of uncertainty for all input parameters, and uncertainty related to the structure of the model and assumptions.	6-8, Table 3 p7
Characterising heterogeneity	21	If applicable, report differences in costs, outcomes, or cost-effectiveness that can be explained by variations between subgroups of patients with different baseline characteristics or other observed variability in effects that are not reducible by more information.	6-8, Table A3-A6
**Discussion**			
Study findings, limitations, generalisability, and current knowledge	22	Summarise key study findings and describe how they support the conclusions reached. Discuss limitations and the generalisability of the findings and how the findings fit with current knowledge.	8-10
**Other**			
Source of funding	23	Describe how the study was funded and the role of the funder in the identification, design, conduct, and reporting of the analysis. Describe other non-monetary sources of support.	11
Conflicts of interest	24	Describe any potential for conflict of interest of study contributors in accordance with journal policy. In the absence of a journal policy, we recommend authors comply with International Committee of Medical Journal Editors recommendations.	11
